# Machine learning based seizure classification and digital biosignal analysis of ECT seizures

**DOI:** 10.1038/s41598-025-88238-3

**Published:** 2025-02-21

**Authors:** Max Kayser, René Hurlemann, Alexandra Philipsen, Nils Freundlieb, Maximilian Kiebs

**Affiliations:** 1 Department of Psychiatry and Psychotherapy, University Hospital Bonn, Bonn, Germany; 2 Department of Psychiatry and Psychotherapy, School of Medicine and Health Sciences, Carl von Ossietzky University of Oldenburg, Oldenburg, Germany; 3 GIB Foundation, Berlin, Germany; 4 Department of Psychiatry and Psychotherapy, University Medical Center Hamburg-Eppendorf, Hamburg, Germany; 5 University of Bonn, Faculty of Medicine, Bonn, Germany

**Keywords:** Medical research, Machine learning, Psychiatric disorders, Depression

## Abstract

While artificial intelligence has received considerable attention in various medical fields, its application in the field of electroconvulsive therapy (ECT) remains rather limited. With the advent of digital seizure collection systems, the development of novel ECT seizure quality metrics and treatment guidance systems in particular will require cutting-edge digital seizure analysis. Using artificial intelligence will offer more analytical degrees of freedom and could play a key role in enhancing the precision of currently available procedures. To this end, we developed the first machine learning (ML) framework that can classify ictal and non-ictal EEG segments, accurately identifying seizure endpoints—a critical step in deriving seizure quality parameters—and computing these metrics at least as reliable as existing precomputed scores. The ML model retained in this study effectively discriminated ictal from non-ictal EEG segments with 89% accuracy, precision, and sensitivity. The reproduced ECT quality parameters showed correlations up to ϱ = 0.99 (*p* < 0.01) with the pre-calculated values from the stimulation device and did not significantly differ from the reference values. Mean seizure duration differences were 0.23 ± 15.59 s compared to the expert rater and 0.28 ± 16.19 s compared to the stimulation device. The study highlights the potential of integrating ML into the field of ECT and emphasizes the critical role of a highly sensitive seizure detection method in reliably determining seizure duration and deriving subsequent quality indices, paving the way for more individualized treatment strategies and novel approaches to determine seizure quality.

## Introduction

### Background

Therapy-refractory major depressive (MDD) and bipolar disorder (BD) are life-shortening illnesses associated with substantial psychological burden and economic costs^1–3^. Most common management strategies include switching or combining antidepressants as well as specific psychotherapeutic interventions^4^. Several treatment guidelines include neurostimulation therapies such as repetitive transcranial magnetic stimulation or electroconvulsive therapy (ECT) as a third approach when other interventions have failed or, in the case of ECT, in the presence of e.g. severe suicidality, psychotic symptoms or catatonia in non-refractory depression^5^. Despite being a well-established treatment associated with high remission rates and improved tolerability as a treatment of MDD as well as schizophrenia^6^, ECT lacks an internationally standardized best-practice^7^. Consequently, there is still a significant gap in scientifically proven recommendations for the implementation of individually tailored ECT series^6^. Much effort has been made to quantify seizure quality and relate it to response to ECT with varying results^8,9^. There is evidence that specific seizure and electroencephalographic (EEG) parameters, as well as changes in physiological measures such as heart rate, may have predictive value for response to ECT treatment^10–12^. Treatment-related predictors concerning the occurrence and duration of cognitive effects after ECT are still limited. Their magnitude has been shown to vary depending on various technical parameters of ECT as well as patient factors^13,14^. For example, a relationship has been found between cognitive impairment and electrical dose, pulse width or amplitude^15–18^.

Research regarding treatment optimization and personalization e.g. addressing the role of individual seizure quality, anaesthesia or dosing is often challenged by small sample sizes, limited characterizations of samples and description of specific treatment details or missing follow-up measures. Large samples and longitudinal data over the entire treatment series including maintenance treatments are rarely published^7^. Further, the scientific understanding of ECT seizure quality and their therapeutic relevance is largely based on pre-calculated EEG seizure quality indices before and after treatment. This deficiency may be partly explained by the fact that data collection of treatment and seizure data has so far mainly been possible by time-consuming manual transfer to external databases. Commercially available ECT devices typically include built-in automated seizure duration determination, which shows moderate to strong correlation with visual determination^19–26^. However, most studies have established reliability using very small sample sizes, and accurate seizure termination is still an issue in routine clinical practice^27^. Studies on the validity of seizure quality indices are sparse, as the relationship between ictal features and clinical outcome is very complex^8^. Currently, there are no widely accepted alternative computerised and open ECT seizure detection methods in clinical practice. Automatic seizure duration detection based on fractal dimension (FD) and entropy-based algorithms have demonstrated good interrater reliability when compared with experienced human raters^28–31^. Still, these algorithms focus solely on seizure endpoint detection, have only been studied in very small samples and are therefore prone to error and noise artefacts. To our knowledge, open and ready-to-use libraries for calculation of seizure quality do not exist.

**Fig. 1:**
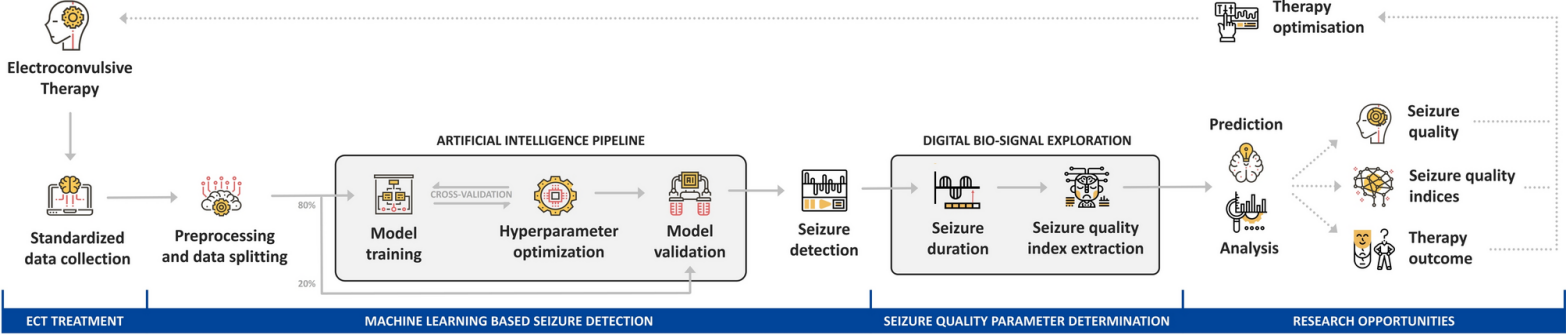
Simplified flowchart including machine learning pipeline for ECT-induced seizure detection, seizure quality index extraction, and resulting research opportunities.

Recent technological advances in automatic digital collection, storage and export of all treatment-data and related biosignals at a patient level pave the way for continuously generated large-scale longitudinal data^32^. As ML has not previously been used for seizure detection in ECT, we developed a machine learning (ML) framework for EEG data from ECT treatments^33^. This study pioneers the use of ML techniques in this context, offering several research opportunities for digital analysis of EEG seizure data (Fig. 1). First, relevance of EEG features for treatment success may yield innovative treatment quality indices. Second, longitudinal analysis could potentially guide procedural decision-making and establishing of treatment algorithms and potentially allowing prediction of treatment success and undesired effects. Yet, real-world EEG seizures and associated treatment parameters are complex multidimensional and unbalanced datasets. ML has emerged as a powerful tool for detecting and classifying patterns within large-scale and highly complex real-world clinical data structures as time- and cost-effectively as possible^34^. ML models can recognize patterns with the help of algorithms and consequently make a prediction or classification for new data. This capability might overcome the limitations of traditional seizure detection methods, offering a more progressive approach. Crucially, as a prerequisite to these types of data-analyses, digital seizure classification and detection is critical since most currently used seizure quality indices are based on a seizure endpoint determination. Lastly, unlike in epilepsy research, the lack of comprehensive references on this topic further highlights the challenge of developing reliable ECT seizure detection methods.

### Objective

Consequently, developed a novel Python-based machine learning and EEG analysis framework to detect ECT induced seizures and to digitally explore seizure brain activity. We hypothesize that this framework can (1) correctly classify seizure segments and non-seizure EEG segments (2) accurately determine the seizures endpoint compared to a reference ECT system and an expert rater (3) calculate seizure parameters at least as accurate as currently available pre-computed scores. Further, we explore five commonly used ML algorithms regarding their classification performance.

## Results

### Dataset and clinical characteristics

The dataset (Table 1) comprised a total of 1634 ECT treatments conducted on 116 patients (75 females, mean age: 59 years, range: 19–90). Patients underwent a minimum of 5 and a maximum of 29 treatments (mean = 14 treatments). 83 patients met the response-criterion. The electrode positions used in the treatment were as follows: bifrontal (30 subjects), bitemporal (10 subjects), unilateral right (70 subjects), unilateral left (5 subjects), and left anterior right temporal (1 subject). The charge delivered during the treatment varied between 25 to 1008 mC, with a mean of 370 mC (SD = 215 mC).

**Table 1:** Dataset characteristics.

	Variable			Total	Responder	Non-Responder	Min	Max	Unit
Clinical characteristics	Patients			116	83	33			$n$
	Sex	Male		75	57	18			$n$
		Female		41	26	15			$n$
	Age			59 (16)	61 (16)	55 (16)	19	90	mean ($SD$)
	Weight			75 (20)	73 (20)	80 (18)	44	176	mean ($SD$), kg
	Diagnosis	Bipolar disorder, current episode depressed	F31.4	8	8	0			$n$
			F31.8	1	1	0			$n$
		Major depressive disorder	F32.2	12	7	5			$n$
			F32.3	8	8	0			$n$
			F33.1	5	4	1			$n$
			F33.2	67	42	25			$n$
			F33.3	14	13	1			$n$
			F33.4	1	1	0			$n$
ECT treatment	Number of ECT sessions			14 (6)	15 (5)	13 (7)	5	29	mean ($SD$), $n$
	Stimulation energy %			73 (42)	71 (41)	78 (44)	5	200	mean ($SD$), %
	Stimulus duration			7 (1)	7 (1)	7 (1)	3	8	mean ($SD$), s
	Charge delivered			370 (215)	360 (210)	396 (226)	25	1022	mean ($SD$), mC
	Electrode position	Bifrontal		30	25	5			$n$
		Bitemporal		10	5	5			$n$
		Left anterior right temporal		5	4	1			$n$
		Unilateral left		1	1	0			$n$
		Unilateral right		70	48	22			$n$

### ML-based ECT seizure detection

**Fig. 2:**
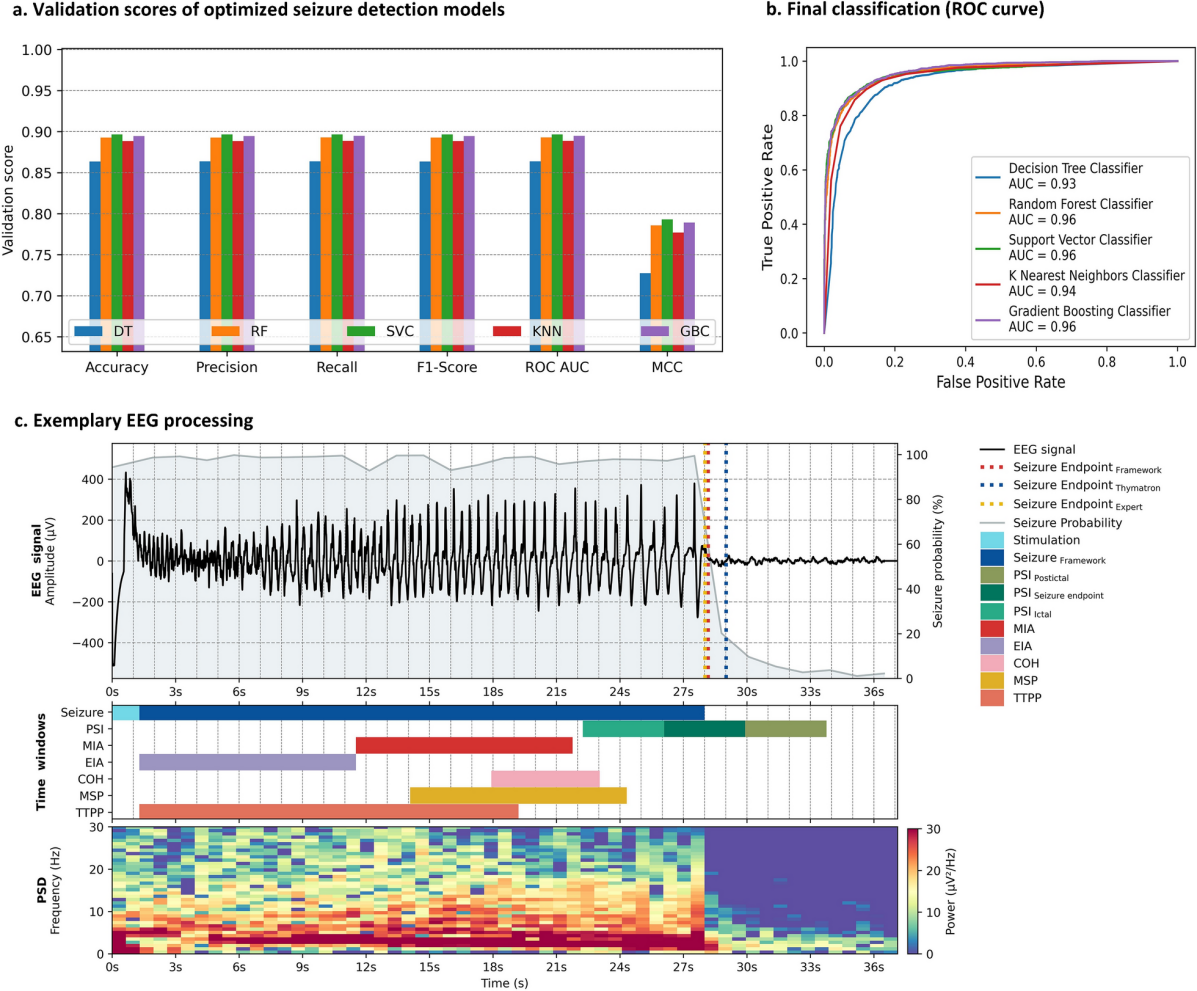
Classification performance and exemplary EEG processing framework. (**a**) Validation metrics of tuned machine learning models. (**b**) ROC curve of optimized classifiers, (**c**) Single channel EEG with ECT inducted seizure, segmented into equally sized time windows with corresponding seizure probabilities. Additionally, were added the experimental (Framework), the precomputed (Thymatron®) as well as the seizure endpoint provided by the expert rater. The subplot below depicts various segments associated with the extraction of seizure quality parameters. The last subplot demonstrates the application of power spectral analysis along the EEG.

The decomposition of the EEG recordings yielded a pool of 38,367 segments, comprising 28,622 ictal segments and 9745 non-ictal segments. Following the initial extraction of 34 features per segment, 17 features were retained after the application of a variance and correlation filter (Appendix B.1). Following undersampling, the dataset consisted of 9745 EEG segments per label, which were subsequently splitted into a training (${n}_{train}=$ 15,592), and a test subset (${n}_{test}=$ 3898). The number of principal components was reduced to 6 through PCA. After conducting hyperparameter optimization with a total of 6705 iterations across all classifiers, the following results were obtained (see Table 2 and Fig. 2 a,b): DT showed uniform performance with 86% accuracy, precision, recall, and F1-Score, complemented by an 86% ROC-AUC and a 0.72 MCC. DT’s peak performance was achieved with a maximum tree depth of 10, a minimum sample split of 8, a minimum samples per leaf of 8, entropy as criterion and the square root of the total number of features set as the maximum number of input features. RF demonstrated an accuracy, precision, recall and F1-Score of 89%, as well as ROC-AUC of 96%, and a MCC of 0.80. The retained hyperparameters for RF encompassed a forest size set to 75, a maximum tree depth of 15, a minimum sample split of 4, a minimum samples per leaf of 3, entropy as criterion and the binary logarithm of the total number of features set as the maximum number of input features. SVC showcased an accuracy, precision, recall, F1-Score and ROC-AUC all at 89%, accompanied by a MCC of 0.79. Notably, the optimal hyperparameters for SVC included a Radial Basis Function kernel, a regularization parameter $C$ set to 10, and a kernel coefficient $\gamma$ equivalent to the reciprocal of the product of n_features_ and the variance of the training data. KNN exhibited consistent performance with 88% accuracy, precision, recall, F1-Score, and ROC-AUC, along with a 0.77 MCC. KNN’s best performing hyperparameters included the number of neighbours set to 7, a Euclidean metric, a distance weighting, and a BallTree algorithm for nearest neighbour calculation. GBC achieved 89% accuracy, precision, recall, and F1-Score, along with an 89% ROC-AUC and a 0.78 MCC. GBC’s classification results were attained with an exponential loss function, 125 boosting stages, a maximum tree depth of 5 and a learning rate of 0.1. The differences in classification performance between ‘ictal’ and ‘non-ictal’ labels, as measured by precision, recall, and F1 metrics, were 1% for RF, SVC, and GBC, 2% for KNN, and 3% for DT.

**Table 2:** Classifiers’ validation scores and comparative results.

			Optimized machine learning classifiers				
			DT	RF	SVC	KNN	GBC
Grid Search	$n$_hyperparameter combinations_		256	768	45	72	200
	$n$_fits_		1280	3840	225	360	1000
Classification metrics	Accuracy		0.86	0.89	0.90	0.89	0.89
	ROC-AUC		0.86	0.89	0.90	0.89	0.89
	MCC		0.73	0.79	0.79	0.78	0.79
	Precision	ictal	0.88	0.90	0.90	0.90	0.90
		non-ictal	0.85	0.88	0.90	0.88	0.89
		*weigthed average*	0.86	0.89	0.90	0.89	0.89
	Recall	ictal	0.85	0.89	0.90	0.88	0.89
		non-ictal	0.88	0.90	0.89	0.90	0.90
		*weigthed average*	0.86	0.89	0.90	0.89	0.89
	F_1_-Score	ictal	0.86	0.89	0.90	0.89	0.90
		non-ictal	0.86	0.89	0.89	0.89	0.89
		*weigthed average*	0.86	0.89	0.90	0.89	0.89
Confusion matrix	Label frequency distribution	TP	1672	1713	1700	1708	1712
		TN	1694	1767	1795	1755	1775
		FP	299	226	198	238	218
		FN	233	192	205	197	193
Model comparison	Cochran’s Q		$Q$= 0.33, *p* = 0.85				
	McNemar’s test	RF	x^2^ = 42.00 *p* = 0.000				
		SVC	x^2^ = 46.68 *p* = 0.000	x^2^ = 1.70 *p* = 0.192			
		KNN	x^2^ = 26.41 p = 0.000	x^2^ = 1.70 *p* = 0.193	x^2^ = 5.72 *p* = 0.017		
		GBC	x^2^ = 42.99 *p* = 0.000	x^2^ = 0.34 *p* = 0.558	x^2^ = 0.50 *p* = 0.480	x^2^ = 3.11 *p* = 0.078

Overall, the classifiers did not differ in their classification performance (Q = 0.33, *p* = 0.85). Pairwise comparisons showed DT classification to perform significantly worse than all other classifiers (x^2^ = 26.41 to 46.68, all *p* ≤ 0.01, see Table 2). KNN classification was significantly worse than SVC (x^2^ = 7.72, *p* ≤ 0.05). All other pairwise comparisons were not significant (all *p* > 0.05). RF classification results were chosen as the basis for seizure endpoint determination in full dataset which in turn was used to calculate the seizure quality indices.

### Seizure quality indices

**Fig. 3:**
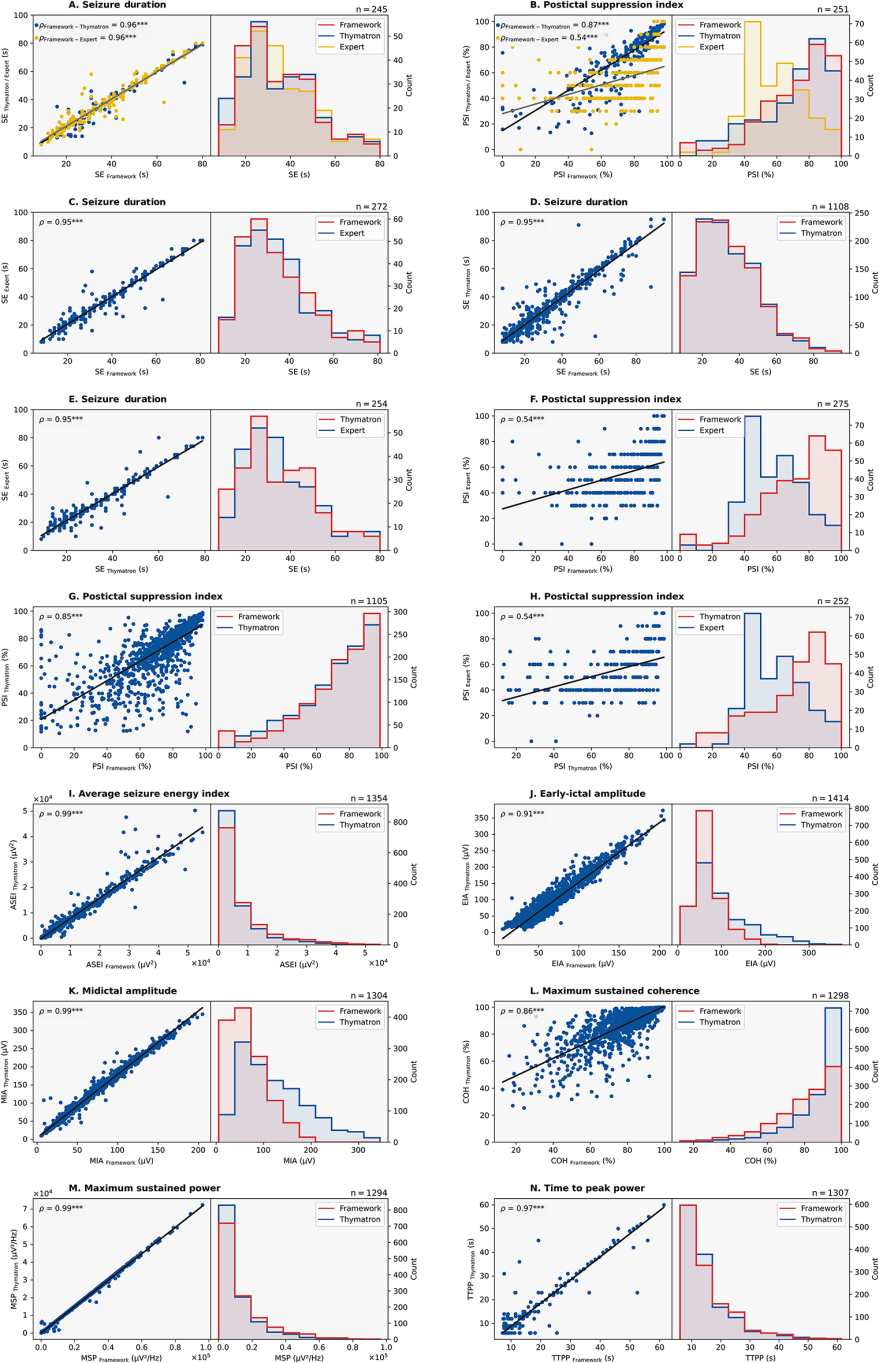
Spearman correlations (left columns) and distributions (right columns) for seizure quality indices calculated by the framework, by the device and rated by the expert. In the subset, rated by the expert: The relationships between the framework, ECT device (Thymatron), and expert ratings for seizure duration (**A**) and PSI (**B**) are shown; the relationship between the expert and the stimulation device is shown for seizure duration (**E**) and for PSI (**H**). Relationships between the expert and the framework are plotted for the seizure duration (**C**) and PSI (**F**). Relationships between the framework and ECT device are plotted for seizure duration (**D**), PSI (**G**), ASEI (**I**), EIA (**J**), MIA (**K**), MSC (**L**), MSP (**M**), and TTPP (**N**).

1634 EEGs were analyzed, and seizure quality measures were calculated by both the framework and the stimulation device (Table 3, Figs. 2c and 3). 305 EEGs were additionally evaluated by an ECT expert in terms of seizure duration and PSI.

**Table 3:** Descriptives and comparison of reference values with expert rater and computed values.

		Subset	$n_{{{\text{subset}}}}$	$\mathop \sum \limits_{NA}$	$Mean$		$SD$	$SEM$	Spearman	MWU
Seizure quality indices	Seizure duration ($s$)	Framework	272	246	34.44	Δ = 0.23	15.59	0.95	ϱ = 0.95 *p* < 0.01	U = 36,543 *p* = 0.81
		Expert		20	34.67		15.71	0.95		
		Framework	1108	246	34.15	Δ = 0.28	16.19	0.49	ϱ = 0.95 *p* < 0.01	U = 617,693 *p* = 0.80
		Thymatron		4	33.87		16.21	0.49		
		Thymatron	254	475	34.57	Δ = 0.52	15.76	0.99	ϱ = 0.95 *p* < 0.01	U = 31,590 *p* = 0.69
		Expert		20	35.09		15.79	0.99		
	PSI (%)	Framework	248	262	69.29	Δ = 14.94	22.61	1.44	ϱ = 0.52 *p* < 0.01	U = 44,580 *p* < 0.01
		Expert		25	54.35		18.04	1.15		
		Framework	1077	262	71.44	Δ = 0.15	21.48	0.65	ϱ = 0.94 *p* < 0.01	U = 584,356 *p* = 0.76
		Thymatron		524	71.28		21.08	0.64		
		Thymatron	252	524	70.09	Δ = 15.72	21.68	1.37	ϱ = 0.54 *p* < 0.01	U = 45,850 *p* < 0.01
		Expert		25	54.37		17.97	1.13		
	ASEI ($\mu V^{2}$)	Framework	1354	195	7940.30	Δ = 1756.98	9177.77	249.42	ϱ = 0.99 *p* < 0.01	U = 1,037,207 *p* < 0.01
		Thymatron		209	6183.33		7487.66	203.49		
	EIA ($\mu V$)	Framework	1414	219	68.79	Δ = 28.09	32.14	0.85	ϱ = 0.91 *p* < 0.01	U = 749,082 *p* < 0.01
		Thymatron		74	96.88		61.68	1.64		
	MIA ($\mu V$)	Framework	1304	253	66.82	Δ = 54.42	39.31	1.09	ϱ = 0.99 *p* < 0.01	U = 428,448 *p* < 0.01
		Thymatron		236	121.24		69.39	1.92		
	COH ($\%$)	Framework	1298	219	79.58	Δ = 8.09	16.77	0.47	ϱ = 0.86 *p* < 0.01	U = 545,874 *p* < 0.01
		Thymatron		242	87.67		13.67	0.38		
	MSP $\left({\mu V^{2}}/{Hz}\right)$	Framework	1294	265	13,302.21	Δ = 3383.23	14,690.34	408.38	ϱ = 0.99 *p* < 0.01	U = 948,803 *p* < 0.01
		Thymatron		234	9918.98		10,973.98	305.07		
	TTPP ($s$)	Framework	1307	219	15.41	Δ = 1.25	8.56	0.24	ϱ = 0.97 *p* < 0.01	U = 949,914 *p* < 0.01
		Thymatron		232	14.16		8.56	0.24		

Subset: considered subset for statistical analysis, ${n}_{subset}$: sample size of the subset used in each comparison, ${\sum }_{NA}$: number of missing values, Mean: arithmetic mean, $SD$: Standard deviation, $SEM$: Standard error of the mean, Levene: Levene ‘s Test of Equality of Error Variances, Spearman: Spearman’s rank correlation coefficient, MWU: Mann–Whitney U-Test.

Sample size variations across comparisons are mainly due to differences in data availability. Not all EEG recordings provided sufficient data for both device and framework calculations, and expert evaluations were limited to a subset due to resource constraints. Missing values in the framework arise from insufficient postictal data (e.g., for PSI computation) or when the ictal portion of the EEG was too short for calculation of metrics like EIA, MIA, and COH. For the Thymatron, missing values may occur if the device failed to identify a seizure endpoint, particularly when the EEG recording ended immediately after a potential endpoint without a postictal phase.

In the EEGs classified by the framework, discernible seizure activity was absent in 131 cases. In 115 cases either the recording ended prematurely, or the available postictal signal was too short for accurate seizure endpoint detection, resulting in a total of 1388 detected seizures. The stimulation device detected 1159 seizures, while no seizure endpoint was provided for 475 EEGs. Regarding the expert rater’s assessment: 285 seizures were observed, 10 EEGs showed no identifiable seizures, and 10 did not meet the minimum duration criterion. In 246 EEGs, no seizure endpoint could be identified by the framework and therefore no seizure quality indices could be determined. Additionally, in 63 instances, the brief duration of seizures rendered calculations for EIA, MIA, and MSP unattainable. In 46 cases, a postictal increase in EEG activity was detected, resulting in a PSI value of 0%. Furthermore, PSI calculation was impeded in 92 cases, as the EEG recording terminated too shortly after seizure conclusion.

Spearman’s rank correlations between seizure duration determined by the framework and the expert rater (ϱ = 0.95, *p* < 0.01) as well the stimulation device (ϱ = 0.95, *p* < 0.01) were very high (Fig. 3.A). Mean differences were negligible (Δ Framework vs. Expert: 0.23 s, *U* = 36,543, *p* = 0.807; Δ Framework vs. stimulation device: 0.28 s, *U* = 617,693, *p* = 0.798). Importantly, it should be noted that all mean seizure durations provided include adequate and inadequate seizures. Framework PSI also strongly correlated with the device calculated values (ϱ = 0.94,* p* < 0.01) and the expert rating (ϱ = 0.52, *p* < 0.01) and yielded a comparable mean value as the stimulation device (both 71%; *U* = 584,356, *p* = 0.761) but deviated by 14.94% from the expert rating (U = 44,580, *p* < 0.01). However, the stimulation device and the expert differed similarly high (Δ = 15.72%, *U* = 45,850, *p* < 0.01), but also correlated strongly on both metrics (seizure duration: ϱ = 0.95, PSI: ϱ = 0.54, *p* < 0.01). Concerning the remaining seizure quality indices, correlations between framework and stimulation device were very strong: 0.99 for ASEI, 0.91 for EIA, 0.99 for MIA, 0.86 for COH, 0.99 for MSP, and 0.97 for TTPP (all *p* < 0.01). Mean differences between the framework and stimulation device were mostly low: ASEI 1756.98 µV^2^, EIA = 28.09 µV, MIA = 54.42 µV, COH = 8.09%, MSP = 3383.23 µV^2^/Hz, and TTPP = 1.25 s, but significant (all *p* < 0.05, see Table 3).

Levene test revealed significant variance differences in PSI between the framework vs. expert and stimulation device vs. expert subsets and indicated significant variance distinctions between the Framework and stimulation device subsets for ASEI, EIA, MIA, COH, and MSP (all *p* < 0.01). Moreover, Shapiro test showed a lack of normal distribution across all calculated and observed seizure quality measures (all *p* < 0.01).

Intraclass correlation coefficients (Table 4) revealed excellent agreement for seizure duration among the framework, stimulation device, and expert, with ICC3 = 0.97 (95% CI 0.96–0.97) and ICC3K = 0.99 (95% CI 0.99–0.99). For the postictal suppression index (PSI), moderate agreement was observed among individual raters (ICC3 = 0.58, 95% CI 0.51–0.64), while averaged ratings demonstrated good reliability (ICC3K = 0.81, 95% CI 0.76–0.84). For other seizure quality indices assessed between the framework and stimulation device, ICC3 values ranged from 0.77 to 0.96, indicating strong reliability across all metrics. COH showed the lowest ICC3 (0.77, 95% CI 0.75–0.79), while MSP demonstrated the highest (0.96, 95% CI 0.95–0.96). Averaged ratings further improved reliability, with ICC3K values ranging from 0.87 to 0.98. Metrics such as MSP and ASEI achieved excellent ICC3K values of 0.98 (95% CI 0.98–0.98), and TTPP showed similarly high reliability (ICC3K = 0.94, 95% CI 0.93–0.94). EIA and MIA also demonstrated strong reliability, with ICC3K values of 0.88 (95% CI 0.86–0.89) and 0.92 (95% CI 0.91–0.93), respectively.

**Table 4:** Intraclass correlation coefficients for seizure quality metrics.

		Type	ICC	95% CI	F-test			
					$F$	df_1_	df_2_	$p$-value
Seizure quality indices	Seizure duration (s)	ICC3	0.97	0.96–0.97	87,58	244	488	*p* < 0.01
		ICC3K	0.99	0.99–0.99	87,58	244	488	*p* < 0.01
	PSI ($\%$)	ICC3	0.58	0.51–0.64	5,16	250	500	*p* < 0.01
		ICC3K	0.81	0.76–0.84	5,16	250	500	*p* < 0.01
	ASEI (${\mu V}^{2}$)	ICC3	0.96	0.95–0.96	47,08	1361	1361	*p* < 0.01
		ICC3K	0.98	0.98–0.98	47,08	1361	1361	*p* < 0.01
	EIA ($\mu V$)	ICC3	0.78	0.76–0.80	8,03	1421	1421	*p* < 0.01
		ICC3K	0.88	0.86–0.89	8,03	1421	1421	*p* < 0.01
	MIA ($\mu V$)	ICC3	0.85	0.84–0.87	12,44	1309	1309	*p* < 0.01
		ICC3K	0.92	0.91–0.93	12,44	1309	1309	*p* < 0.01
	COH ($\%$)	ICC3	0.77	0.75–0.79	7,76	1303	1303	*p* < 0.01
		ICC3K	0.87	0.86–0.88	7,76	1303	1303	*p* < 0.01
	MSP $\left({\mu V^{2}}/{Hz}\right)$	ICC3	0.96	0.95–0.96	46,56	1299	1299	*p* < 0.01
		ICC3K	0.98	0.98–0.98	46,56	1299	1299	*p* < 0.01
	TTPP ($s$)	ICC3	0.88	0.87–0.89	15,98	1312	1312	*p* < 0.01
		ICC3K	0.94	0.93–0.94	15,98	1312	1312	*p* < 0.01

ICC: Intraclass correlation coefficient, ICC3: Intraclass correlation coefficient among individual raters, ICC3K: Intraclass correlation coefficient averaged ratings across raters, F-test ($F$): F-statistic, df_1_/df_2_: Degrees of freedom, $p$-value: Level of significance, 95% CI 95% Confidence interval.

## Discussion

This bicentric real-world data collection demonstrated once again the substantial heterogeneity among the individuals undergoing ECT and within the treatment procedure itself. Variability across a spectrum of variables, including the number of treatment sessions, demographic variables such as age and sex, clinical variables like diagnosis and treatment outcomes, as well as technical aspects such as electrode positions, and charge delivered. However, with novel methods of longitudinal data collection systems being implemented into modern ECT systems, the existing challenges of how stimulus parameters, seizure quality and dosing relate to the individual treatment efficacy and acceptability may be viewed in a new light. Specifically, novel seizure quality metrics and treatment guidance systems will depend on cutting-edge digital seizure analysis and classification models. Hence, we developed a machine-learning based framework which has shown to be highly accurate in distinguishing seizure segments from non-seizure segments and can form the basis for calculating commonly used ECT seizure quality metrics.

In this study, the DT model displays overall reliability, it falls short in comparison, revealing a slightly lower MCC and heightened rates of false positives (FP) and false negatives (FN), suggesting greater vulnerability to misclassifications than other classifiers. The RF closely follows, showcasing improvements over the DT, with a respectable MCC and accuracy. Its F1-Scores for both labels reflect a balanced performance in terms of precision and recall. The SVC stands out with the highest MCC, accuracy and F1-score, showcasing superior precision and recall for both classes and striking a commendable balance in minimizing FP and FN. While the KNN and GBC deliver competitive MCCs results, they slightly trail behind SVC and RF in terms of accuracy and overall model performance. RF, SVC, and GBC, distinguished only by minimal variances in class-related performance, may thereby hold promise for reducing FN results. Lack of statistically significant heterogeneity among the classifiers supports the notion of comparable well-balanced predictive performance across all classifiers, suggesting that the choice between classifiers may not significantly impact predictive outcomes. Pairwise comparisons did reveal significant differences particularly of DT against all other classifiers and the KNN versus SVC comparison yielded a disagreement between the two classification methods. However, after reviewing the evaluation metrics and statistical model comparison, it can be concluded that all classifiers demonstrate a comparable overall performance. While DT appears to be somewhat less reliable in the classification of ECT-induced seizures, SVC and RF exhibit the utmost and well-balanced performances among classifiers, making them the most favourable choices.

In addition to the models’ excellent classification performance of seizure activity and endpoint, another, less obvious type of performance should be evaluated. The capability of modelling without producing missing values, which, in clinical routine, is challenging even for the stimulation device itself^27,35^. However, interpreting the number of missing values for seizure parameter estimation by the framework proved challenging for several reasons. First, if the original dataset is not rated by an expert, it may theoretically be possible for the stimulation device to identify seizures even when none are present, leading to false model input. The used dataset may therefore consist of instances where the device has accurately and inaccurately determined the presence or absence of seizures. Hence, the framework’s inability to calculate a seizure endpoint can either arise from the fact that it was unable to detect a seizure or was just not able to compute whether a seizure occurred even if it had occurred. Second, as even a slight deviation in the calculated seizure endpoint may result in a situation where the ictal or postictal EEG signal is technically too brief to calculate quality parameters. Thus, in the absence of large datasets evaluated by an expert rater, the framework’s performance to robustly produce non-missing values, can only be compared across all seizures, regardless of whether it was a FN from the stimulation device or a true positive (TP) from the framework. Given the goal of this study to reconstruct and validate established seizure parameters, the resulting overall number of unsuccessful calculations may be considered acceptable. In the context of seizure detection, the ML pipeline exhibited notable efficacy when compared to the ECT device and expert evaluations. Specifically, the framework detected seizures in 85% of EEGs, a significant improvement over the stimulation devices’ detection rate of 71%. In comparison, expert evaluations achieved the highest detection rate, identifying seizures in 93% of cases. These findings underscore the promising performance of the framework, positioning it as a robust contender in seizure detection, albeit with distinctions from both Thymatron and expert assessments. Still, without large datasets rated by an expert, the true classification performance cannot be ascertained. Further exploration and validation of the framework’s capabilities are warranted to elucidate its potential as a valuable tool in clinical EEG analysis.

The approach aligns well with the ECT device for most seizure quality indices, demonstrating minimal discrepancies in mean differences and suggesting reliable reproduction of established markers. The seizure duration measurements were highly consistent with both expert evaluations and the device readings, supported by strong intraclass correlation. It should be acknowledged that the mean seizure duration accounts for inadequate seizures as well as adequate seizures, resulting in lower values compared to the average duration of typical seizures. Significant differences were observed for the PSI when compared to expert assessments. The PSI’s sensitivity to precise seizure endpoint detection emphasizes the critical need for accurate seizure detection to ensure reliable PSI calculations and effective treatment assessment. Minor inaccuracies in determining the seizure endpoint can have a substantial impact on PSI values. While the PSI showed moderate agreement among individual raters, averaging ratings across methods improved consistency, as reflected in the higher ICC3K value. Other indices, such as COH, MSP, and EIA, demonstrated strong agreement, further validating the framework’s ability to accurately capture key seizure characteristics. MIA and TTPP also showed good agreement, though some variability may stem from challenges in assessing mid-seizure dynamics and time-sensitive metrics. These results highlight the framework’s robustness in providing reliable seizure assessments across multiple indices, reinforcing its potential to support clinical decision-making.

Our conceptual framework represents an unprecedented advancement in the field of ECT by integrating ML techniques for seizure detection alongside data transformation, feature extraction, dimensionality reduction, and digital analysis of EEG seizures. This innovative approach introduces novel methodologies that significantly enhance our ability to analyze and interpret ECT-induced seizures. Future research should make use of the extracted features and associated feature importance’s to assess their potential relevance for treatment success and undesired effects minimization. This research holds the promise of not only understanding the dynamics of seizures but also tailoring ECT interventions to individual patient needs. This might be achieved by the development of alternative seizure quality markers, leveraging ML to identify novel indicators that may offer more nuanced insights into the therapeutic impact of ECT. Additionally, alternative seizure indices or predictive biomarkers could significantly outperform conventional EEG assessments and might potentially lead to machine-learning based procedural guidance tools (e.g. individualized dosing recommendations). In addition, supplementing current treatment systems with novel analytic frameworks that use not only novel seizure indices, but also sociodemographic and treatment-related variables, may help to focus attention on the cause and possible resolution of treatment-related problems, artefacts, or user errors to ensure that treatment is applied as accurately and effectively as possible.

As the digitalisation and personalisation of medical treatments, large data sets and related analytics are already underway in many medical fields, digital frameworks may be paving the way for the digital future of ECT, which should in turn improve clinical decision-making and individualise treatments.

The study highlights the potential of integrating ML into the realm of ECT, emphasizing the crucial role of a highly sensitive seizure detection method in reliably determining seizure duration and deriving subsequent quality indices. The study brings attention to the largely successful reproducibility of established and widely used seizure quality indices in clinical seizure assessment, paving the way for innovative seizure markers. Despite a limited number of AI-associated ECT-studies primarily focused on neuroimaging data, the potential integration of AI, along with the availability of highly standardized treatment data from comprehensive multicentre ECT databases such as GENET^32^, holds significant promise in advancing the field of ECT. Continuous research and development efforts have the capability to unveil new possibilities for personalized treatment approaches, empowering clinicians to tailor therapeutic strategies on an individual basis and forecast ECT treatment progress and outcomes. Ultimately, the outlined method also offers an avenue for the future development of a novel, ready-to-use pipeline for accurate ECT-seizure detection and comprehensive digital EEG analysis in clinical or scientific settings.

## Methods

### Study participants

All inpatients (109 MDD and 7 BD) receiving an ECT index course at either the University Hospital Bonn (BN) or University Hospital Hamburg-Eppendorf (HM) were consecutively included in this retrospective study of anonymized medical records. Diagnosis was established based on ICD-10 criteria (see Table 1).

### Electroconvulsive therapy

All participants underwent ECT between 2019 and 2022, with each treatment series consisting of 5 to 29 single sessions. Both sites used a Thymatron® IV (Somatics LLC, Lake Bluff, Illinois, USA) and delivered brief-pulse square-waved stimuli to unilateral or bilateral electrode placements (Table 1). Seizure threshold was determined through dose-titration (BN), or age-method (HM) and treatment interval varied between two (BN) to three (HM) sessions per week. General anaesthesia was typically induced using propofol (1–2 mg/kg) or 1:1 ketamine-propofol admixture (1–2 mg/kg). Subsequent muscle relaxation by administration of succinylcholine or rocuronium bromide. Additional medication was allowed according to international treatment guidelines. Electrocardiogram, blood pressure and brain activity were continuously monitored to assess induced seizures. At the beginning of each ECT session, Clinical Global Impression was evaluated (CGI-S)^36^. Patients who had experienced a decrease of ≥ 2 points from baseline or achieved a CGI-S score of ≤ 3 were deemed responders^37^.

### Data collection

Systematic collection of standardized longitudinal data was done using the ECT data collection tool GENET-GPD^32^. The retrospective dataset comprised various treatment variables, sociodemographic characteristics, biosignals (EEG, ECG), and device-calculated seizure quality indices (e.g., seizure duration and Postictal Suppression Index [PSI]). Latter were calculated by the ECT stimulation device using a two-channel fronto-mastoidal EEG recording with a 50 Hz notch filter and a 30 Hz lowpass filter applied. Seizure endpoint and PSI were also determined by a blinded expert rater, adding an extra layer of validation to the primary device-precomputed values. PSI was scored by the expert in 10% increments, ranging from 0% (no suppression) to 100% (maximal suppression). Seizure duration was scored in seconds. In cases of restimulation, only data from the last stimulation within each respective session was included. Regarding the visual determination of the seizure endpoint, the expert rater focused on the cessation of high-frequency spike-wave complexes and the transition to postictal suppression, a period of low-voltage mixed-frequency activity or flat EEG. The cut-off for seizure termination was defined as the point at which the high-frequency activity diminished and gave way to the postictal suppression phase, signalling the conclusion of the seizure.

### Data processing

The dataset was imported into a scientific Python environment^38^. To effectively manage and process the multidimensional and nested data structure, various core libraries were employed, as detailed in Appendix A. Missing clinical data underwent imputation using last observation carried forward and backward for diagnosis, age, sex, and electrode position along with linear interpolation for CGI. A random seed was set to achieve consistency and reproducibility of the succeeding computations. Single channel 200 Hz EEG recordings were partitioned into non-overlapping segments of 1.28 s each, with the resulting window length providing high temporal resolution to capture rapid changes and transient seizure activity, while considering the lower frequency resolution. Recordings longer than 2 min were excluded (n_seizures_ = 8).

### ML-based ECT seizure detection

Each extracted EEG segment was labelled either as ‘ictal’ or ‘non-ictal’, depending on the reference seizure endpoint determined by the treatment device. Seizures without device determined endpoint were omitted. Segments occurring before or during stimulation were omitted. EEG-relevant features, including signal characteristics such as spectral components (frequency, energy) and statistical moments (mean, variance, skewness, kurtosis), were extracted using the TSFEL^39^ toolbox and investigated with descriptive statistics (Appendix B.1). Low variance (≤ 20%) and highly correlated features (≥ 95%) were eliminated. All remaining features were subsequently balanced out by random undersampling and split into a training and a test subset in a ratio of 8:2. Min–max normalization was applied for feature scaling, followed by principal component analysis (PCA), with a target variance of 80%. Subsequently, five different machine learning algorithms from $Scikit$-$learn$^40^ library including Random Forest (RF), Decision Tree (DT), Support Vector Classifier (SVC), k-Nearest Neighbors (KNN), and Gradient Boosting Classifier (GPC) were used for binary classification of the EEG segments (Appendix B.2). fivefold cross-validated grid-search was implemented to optimize relevant hyperparameters, mitigating the risk of overfitting, with the Matthews Correlation Coefficient (MCC)^41,42^, serving as primary scoring metric. Afterwards, individual predictive capability of each fine-tuned classifier was assessed on the hold-out test set using the ML metrics accuracy, precision, recall, F1-score, MCC, and area under the receiver operating characteristic curve (ROC-AUC; Appendix B.3)^43^. All classifiers were compared comprehensively using a Cochrane Q test^44^ and dichotomous assessments were conducted post hoc using the McNemar test^45,46^. The algorithm with the highest overall performance was employed in the last stage to predict the seizure probability for each EEG segment across all segments. The classifier’s hyperparameters were selected in accordance with the findings of the prior step.

The onset of an ECT induced seizure was deduced when the classification probability for the ‘ictal’ label surpassed the 50% threshold after stimulation for at least 8 s. Conversely, if the seizure probability did fall below 30% for three successive EEG segments, no further convulsive activity was presumed, and the first non-ictal epoch was considered as seizure endpoint. In case there was a recurrence of seizure activity within a 4-s post-ictal window after the determined seizure endpoint and the conditions for a seizure in the subsequent EEG readings were met once again, the latter seizure endpoint would be retained.

### Seizure quality indices

Using the previously established seizure endpoints, we proceeded to determine several ECT seizure quality indices. They were calculated in a manner comparable to those of the stimulation device^47^ (see Appendix C), with the latter serving as the primary statistical reference values (ground truth). Missing values were omitted by list-wise deletion. Seizure duration was then calculated by the elapsed time between seizure onset and conclusion. A segmental power spectrum analysis across multiple frequency bands ($\delta$: 0.7–3.5 Hz; $\theta :$ 3.5–8 Hz; $\alpha$: 8–13 Hz; $\beta$: 13–25 Hz) was carried out using the SciPy^48^ fast Fourier transform method with a Hanning window function and non-overlapping segments^48^. The Average Seizure Energy Index (ASEI) was obtained by dividing the total ictal EEG power by the seizure duration estimated by the pipeline. Maximum Sustained Power (MSP) denotes the peak cumulative power spectral density (PSD) observed within 8 consecutive ictal EEG segments. The time interval between seizure onset and the highest EEG power is referred to as Time To Peak Power (TTPP). The Early-Ictal Amplitude (EIA) corresponds to the average absolute amplitude during the initial phase of a seizure, while the Mid-Ictal Amplitude (MIA) results from the maximum average amplitude observed among any eight sequential seizure segments. The Post-Ictal Suppression Index (PSI) quantifies the transition from seizure-related spike-wave patterns to a subdued non-convulsive signal following seizure termination. It is calculated by comparing the average amplitudes of three postictal EEG segments with those of three seizure segments, while excluding a 4-s time window around the seizure endpoint. The measure of interhemispheric synchronization, referred to as Maximum Sustained Coherence (COH), was determined as the highest mean signal coherence between both EEG channels over a 10-s ictal interval.

Distribution and variance of collected and computed seizure quality indices were investigated using Shapiro–Wilk test^49^ and Levene’s test^50^. Spearman’s rank correlation coefficient^51^ and Mann–Whitney-U-Test^52^ (MWU) were computed among calculated seizure quality scores and both expert ratings (seizure duration and PSI only) and pre-computed values of the stimulation device. Statistical significance was assumed at α < 0.01. Intraclass correlation coefficients (ICCs) were calculated using a two-way mixed-effects model to assess the reliability of seizure quality indices between the framework and the ECT device, with the expert included for seizure duration and PSI. ICC3 evaluated individual rater agreement, ICC3K assessed averaged ratings, and missing values were excluded pairwise. Statistical significance (α < 0.01) was determined using the F-test statistic.

## Supplementary Information

Supplementary Information.

## Data Availability

The datasets analysed in the current study are not publicly available due to compliance to legal requirements but are available from the corresponding author on reasonable request with permission from the University Hospital Bonn.

## Code availability

The underlying code for this paper was developed using open-source libraries listed in Appendix A. The code for calculating digital ECT seizure quality indices has been organized into a Python toolbox called “ECTMetrics”^53^, which is publicly available on GitHub: https://github.com/maxkayser/ectmetrics/. All other code may be made available to qualified researchers upon reasonable request from the corresponding author.
