# Supplementary material for: Machine learning based seizure classification and digital biosignal analysis of ECT seizures
Source: Sci Rep. 2025 Feb 21;15:6409. doi: 10.1038/s41598-025-88238-3 (PMC11845479; doi:10.1038/s41598-025-88238-3)
Supplement: Supplementary file 1 — Supplementary Information. [file 41598_2025_88238_MOESM1_ESM.pdf]

Appendices

A. Python libraries employed

Table S1
Employed Python libraries, including release version, author(s), and their respective usage.

Python library	Version	Author(s)	Usage
ECTMetrics	1.0.1	Kayser et al., 2025[1]	ECT-EEG metrics analysis library developed
imbalanced-learn	0.9.1	Lemaître et al., 2017 [2]	Class imbalance handling
Joblib	1.1.0	Joblib Development Team, 2020 [3]	Parallel computing and caching
matplotlib	3.5.1	Hunter, 2007 [4]	Graphical representation and analysis
MLxtend	0.21.0	Raschka, 2018 [5]	Model performance comparison
NumPy	1.21.6	Harris et al., 2020 [6]	Numerical operations and array manipulation
Pandas	1.3.5	McKinney, 2010 [7]; Reback et al., 2021 [8]	Structured tabular data processing and analysis
Pingouin	0.5.5	Vallat, 2018 [9]	Intraclass correlation coefficients
pyEDFlib	0.1.30	Nahrstaedt et al., 2020 [10]	Bio-signal import and manipulation
Scikit-learn	1.0.2	Pedregosa et al., 2011 [11]	Machine learning toolkit
SciPy	1.7.3	Virtanen et al., 2020 [12]	Numerical and scientific computing
TSFEL	0.1.4	Barandas et al., 2020 [13]	EEG features extraction

References

1. Kayser, M., Hurlemann, R., Philipsen, A., Freundlieb, N. & Kiebs, M. Machine learning based seizure classification and digital biosignal analysis of ECT seizures. Scientific Reports (in press) doi: 10.1038/s41598-025-88238-3

2. Lemaître, G., Nogueira, F. & Aridas, C. K. Imbalanced-learn: A Python Toolbox to Tackle the Curse of Imbalanced Datasets in Machine Learning. Journal of Machine Learning Research **18**, 1–5 (2017).

3. Joblib Development Team. Joblib: running Python functions as pipeline jobs. (2020).

4. Hunter, J. D. Matplotlib: A 2D Graphics Environment. Comput. Sci. Eng. **9**, 90–95 (2007).

5. Raschka, S. MLxtend: Providing machine learning and data science utilities and extensions to Python's scientific computing stack. Journal of Open Source Software **3**, 638 (2018).

6. Harris, C. R. et al. Array programming with NumPy. Nature 585, 357–362 (2020).

7. McKinney, W. Data Structures for Statistical Computing in Python. Proceedings of the 9th Python in Science Conference 56–61 (2010) doi:10.25080/Majora-92bf1922-00a.

8. Reback, J. et al. pandas-dev/pandas: Pandas 1.3.5. Zenodo doi.org/10.5281/ZENODO.5774815 (2021).

9. Vallat, R. Pingouin: Statistics in Python. Journal of Open Source Software 3, 1026 (2018) doi:10.21105/joss.01026.

10. Nahrstaedt, H. et al. holgern/pyedflib: v0.1.23. Zenodo doi.org/10.5281/ZENODO.5678481 (2020).

11. Pedregosa, F. et al. Scikit-learn: Machine Learning in Python. Journal of Machine Learning Research **12**, 2825–2830 (2011).

12. Virtanen, P. et al. SciPy 1.0: fundamental algorithms for scientific computing in Python. Nat Methods **17**, 261–272 (2020).

13. Barandas, M. et al. TSFEL: Time Series Feature Extraction Library. SoftwareX **11**, 100456 (2020).

B. Machine Learning Approach

B.1 Features

The features listed in Table S2 were extracted from each EEG segment using the $TSFEL$ library [1], capturing key statistical and spectral characteristics. These features were then selected based on variance and correlation thresholds (Table S3) before being passed to the classification pipeline.

Table S2
Feature definitions

Feature	Definition
Absolute energy	The absolute energy of a signal is a measure of its total energy, calculated as the sum of the squared magnitudes of all the data points in the signal.
Area under the curve	The area under the curve delineates the total 'area' enclosed by the signal curve, and it is computed as the integral of the signal over a specified time interval.
Autocorrelation	Autocorrelation measures the similarity between a signal and a time-shifted version of itself. It is calculated by convolving the signal with its time-reversed version.
Centroid	The centroid represents the center of mass of the signal's frequency spectrum, reflecting the balance point of spectral energy.
Entropy	The entropy of a signal, based on Shannon's information theory, quantifies the degree of 'randomness' in the signal by evaluating the distribution of its amplitude values.
Fundamental frequency	The fundamental frequency is the lowest frequency component of a periodic signal. It is often associated with the dominant oscillatory rhythm in EEG signals.
Kurtosis	Kurtosis is a statistical metric that measures the degree of 'tailedness' exhibited by a probability distribution.
Max / Maximum	The minimum value of a signal represents the highest amplitude within the signal.
Max power spectrum	The maximum power spectrum refers to the highest value in the power spectral density of the EEG signal, representing the frequency with the most significant energy contribution.
Maximum frequency	The maximum frequency identifies the frequency component with the highest amplitude.
Mean	The mean is the average value of a signal, calculated as the sum of all data points divided by the number of data points.
Mean absolute deviation	The mean absolute deviation measures the average absolute difference between each data point and the mean.
Mean absolute diff	The mean absolute difference typically refers to the average absolute difference between pairs of data points in a signal.
Mean difference	The mean difference is the average of the absolute differences between consecutive data points in the signal.
Median	The median is the middle value of a sorted dataset.
Median absolute deviation	The mean absolute deviation measures the average absolute difference between individual data points and the mean value.
Median absolute diff	The median absolute difference refers to the median of the absolute differences between pairs of data points in a signal.
Median diff	The median difference measures the median value of the differences between adjacent data points in a signal
Median frequency	The median frequency is the frequency at which half of the signal's energy is above it and half is below it.
Min / Minimum	The minimum value of a signal represents the lowest amplitude within the signal.
Negative turning points	Negative turning points is defined by the number of times the signal changes from decreasing to increasing.
Neighbourhood peaks	Neighborhood peaks count the number of local maxima within a specified range.
Peak to peak distance	Peak-to-peak distance measures the time or interval between two successive maxima in a signal.
Positive turning points	Positive turning points refer to the number of times the signal changes from increasing to decreasing.
Power bandwidth	The power bandwidth represents the frequency range that contains a specified percentage of the total signal power.
Root mean square	The root mean square is a measure of the signal's root mean square amplitude.
Signal distance	The signal distance is determined by the total distance travelled, calculated as the hypotenuse between two adjacent datapoints.
Skewness	Skewness measures the asymmetry of a probability distribution.

Slope	The spectral slope quantifies the rate at which power decreases as frequency increases in the signal's spectrum.
Standard deviation	The standard deviation measures the dispersion or variability of a signal's data points.
Sum absolute diff	The sum absolute difference is the sum of the absolute differences between pairs of data points in a signal.
Total energy	Total energy represents the total energy content of a signal, often calculated as the sum of the squared values.
Variance	The variance quantifies the spread or dispersion of data points in a signal.
Zero crossing rate	The zero crossing rate represents the frequency at which the signal crosses the zero amplitude level.

Table S3

Descriptive statistics and feature selection

Feature	Minimum	Maximum	Mean	SD	Variance	Retained
Absolute energy	1.00	$4.70 * 10^7$	$1.18 * 10^6$	$2.12 * 10^6$	< 0.20	Yes
Area under the curve	$8.00 * 10^{-2}$	$5.16 * 10^2$	$5.17 * 10^1$	$4.92 * 10^1$	< 0.20	Yes
Autocorrelation	1.00	$4.70 * 10^7$	$1.18 * 10^6$	$2.12 * 10^6$	< 0.20	No
Centroid	$1.00 * 10^{-2}$	1.24	$6.20 * 10^{-1}$	$1.20 * 10^{-1}$	≥ 0.20	No
Entropy	0.00	1.00	0.93	0.10	≥ 0.20	No
Fundamental frequency	0.00	$5.04 * 10^1$	2.12	1.15	< 0.20	Yes
Kurtosis	-3.00	$2.51 * 10^2$	0.12	2.50	< 0.20	Yes
Max	$-1.19 * 10^1$	$5.12 * 10^2$	$1.23 * 10^2$	$1.16 * 10^2$	< 0.20	Yes
Max power spectrum	0.00	1.10	0.42	0.21	≥ 0.20	No
Maximum frequency	0.00	$9.53 * 10^1$	$5.10 * 10^1$	$1.58 * 10^1$	< 0.20	Yes
Mean	$-1.40 * 10^2$	$1.44 * 10^2$	0.42	6.78	< 0.20	Yes
Mean absolute deviation	0.00	$3.99 * 10^2$	$4.05 * 10^1$	$3.87 * 10^1$	< 0.20	No
Mean absolute diff	0.00	$1.27 * 10^2$	7.85	8.56	< 0.20	Yes
Mean diff	-4.01	3.93	$-1.00 * 10^{-2}$	$3.80 * 10^{-1}$	≥ 0.20	No
Median	$-2.26 * 10^2$	$2.72 * 10^2$	-3.78	$1.43 * 10^1$	< 0.20	Yes
Median absolute deviation	0.00	$4.49 * 10^2$	$3.39 * 10^1$	$3.38 * 10^1$	< 0.20	No
Median absolute diff	0.00	$1.03 * 10^2$	5.83	6.02	< 0.20	No
Median diff	$-2.86 * 10^1$	7.88	$-3.00 * 10^{-1}$	1.59	< 0.20	Yes
Median frequency	0.00	$4.96 * 10^1$	8.01	3.80	< 0.20	Yes
Min	$-5.12 * 10^2$	8.56	$-9.94 * 10^1$	$8.53 * 10^1$	< 0.20	Yes
Negative turning points	0.00	$5.20 * 10^1$	$2.00 * 10^1$	6.20	< 0.20	Yes
Neighbourhood peaks	0.00	$1.80 * 10^1$	6.42	2.16	< 0.20	Yes
Peak to peak distance	0.00	$1.02 * 10^3$	$2.23 * 10^2$	$1.96 * 10^2$	< 0.20	No
Positive turning points	0.00	$5.30 * 10^1$	$1.99 * 10^1$	6.21	< 0.20	No
Power bandwidth	0.00	$6.64 * 10^1$	$1.37 * 10^1$	7.65	< 0.20	Yes
Root mean square	$6.00 * 10^{-2}$	$4.29 * 10^2$	$4.98 * 10^1$	$4.61 * 10^1$	< 0.20	No
Signal distance	$2.55 * 10^2$	$3.23 * 10^4$	$2.07 * 10^3$	$2.15 * 10^3$	< 0.20	No
Skewness	$-1.59 * 10^1$	$1.59 * 10^1$	$1.10 * 10^{-1}$	$6.10 * 10^{-1}$	< 0.20	Yes
Slope	-1.66	2.34	$-1.00 * 10^{-2}$	$1.20 * 10^{-1}$	≥ 0.20	No
Standard deviation	0.00	$4.23 * 10^2$	$4.95 * 10^1$	$4.60 * 10^1$	< 0.20	No
Sum absolute diff	0.00	$3.23 * 10^4$	$2.00 * 10^3$	$2.18 * 10^3$	< 0.20	No
Total energy	$7.80 * 10^{-1}$	$3.69 * 10^7$	$9.25 * 10^5$	$1.66 * 10^6$	< 0.20	No
Variance	0.00	$1.79 * 10^5$	$4.56 * 10^3$	$8.19 * 10^3$	< 0.20	No
Zero crossing rate	0.00	$7.00 * 10^1$	$1.57 * 10^1$	8.69	< 0.20	Yes

B.2 Machine learning algorithms

A comparative analysis of five different machine learning classifiers from the `Scikit-learn` [2] library was conducted to address the classification task for ECT induced seizure detection, with the hyperparameters of each classifier altered to optimize performance and accuracy.

B.2.1 Decision Tree Classifier

The Decision Tree (DT) classifier operates by recursively partitioning the feature space based on input feature values. It selects the most discriminative feature to split the data at each node, with the goal of minimizing impurity. This process generates a tree-like structure where each path from the root to a leaf corresponds to a decision rule. During prediction, a data point navigates the tree from the root to a leaf, and its class label is determined by the most prevalent class among training instances in that leaf. As part of this study, the performance of the DT algorithm was evaluated by employing varying maximum depth (unlimited, 5, 10 and 15), minimum sample split size (6, 7, 8 and 9), minimum samples per leaf node (5, 6, 7 and 8), maximum input feature count (square root and binary logarithm of $n_{features}$) and splitting criteria (gini impurity and entropy).

B.2.2 Random Forest Classifier

The Random Forest (RF) classifier combines the outputs of multiple individual decision trees. Each tree is trained on a different subset of the training data, obtained through bootstrapping. The final prediction is determined by gathering the predictions of these trees through a majority voting mechanism. Within this research, the RF was examined with variations in the number of trees (75, 100, 125 and 150), maximum depth (unlimited, 5, 10 and 15), maximum number of input features (square root and binary logarithm of $n_{features}$), minimum numbers of leaf nodes samples (1, 2 and 3), splitting criteria (gini impurity and entropy) and the minimum number of samples required to split an internal node (2, 4, 6 and 8).

B.2.3 Support Vector Classifier

The Support Vector Classifier (SVC), closely related to the Support Vector Machine, seeks to classify data points into different classes while striving to achieve a clear margin between the classes. It identifies a hyperplane that optimally separates data points of distinct classes in the feature space. It seeks to maximize the margin between the hyperplane and the nearest data points, known as support vectors. Through kernel functions, SVC can effectively handle non-linear patterns by projecting data into a higher-dimensional space. The choice of kernel (radial basis function kernel, polynomial kernel, and linear kernel), gamma coefficient (reciprocal value of the number of features, reciprocal of the product of $n_{features}$ and the variance of the training data, 0.001, 0.003 and 0.005) and regularization parameter C (0.1, 1 and 10) influences the model's performance and the trade-off between maximizing the margin and minimizing classification errors.

B.2.4 K-Nearest Neighbors classifier

The k-Nearest Neighbors (KNN) classifier makes predictions based on the similarity between a given data point and a given number of nearest neighbors in feature space. The predominant class among these neighboring points determines the final predicted class. In the case of the KNN classifier, evaluations were performed using distinct numbers of neighbors (4, 5, 6, 7, 8 and 9), varying voting weights (uniform and distance based), various distance metrics (euclidean, manhattan and chebyshev), and diverse algorithms for computing neighbors, including BallTree and K-Dimensional Tree.

B.2.5 Gradient Boosting Classifier

The Gradient Boost Classifier (GBC) functions iteratively by combining the outputs of sequentially trained weak learners. Each weak learner, often a shallow decision tree, is fitted to the residual errors of the previous ensemble's predictions. This gradual correction process leads to the gradual improvement of the models' predictive performance. The learning rate parameter controls the contribution of each weak learner to the final prediction. By continually emphasizing previously misclassified instances, the model adapts and becomes more accurate over iterations. As covered by this analysis, the GBC underwent assessments with varying learning rates (0.01, 0.1, 0.2, and 0.3),

maximum depths (3, 4, 5, 6 and 7), the number of boosting stages (50, 75, 100, 125, and 150), and different loss functions (exponential and deviance).

B.3 Machine learning metrics

B.3.1 Confusion matrix

A confusion matrix is a table that summarizes the model's predictions against actual class labels. It contains counts of true positives (TP), true negatives (TN), false positives (FP), and false negatives (FN).

Table S4
Confusion matrix

		Predicted class	
		Positive	Negative
Actual class	Positive	*True Positives*	*False Negatives*
	Negative	*False Positives*	*True Negatives*

B.3.2 Accuracy

Accuracy is a performance metric that quantifies the proportion of the correctly predicted instances among all instances in a dataset, offering a broad assessment of a model's overall performance. The formula for accuracy is:

Eq. (B.3.2) $$Accuracy = \frac{True\ Positives + True\ Negatives}{Total\ Instances}$$

B.3.3 Specificity

Specificity measures the models' ability to correctly identify negative instances. It is the ratio of true negative predictions to the total actual negative instances. The formula for specificity is:

Eq. (B.3.3) $$Specificity = \frac{True\ Negatives}{True\ Negatives + False\ Positives}$$

B.3.4 Recall / Sensitivity

Recall or sensitivity quantifies the ability of a model to identify all positive instances correctly. It is the ratio of true positive predictions to the total actual positive instances. The formula for recall is:

Eq. (B.3.4) $$Recall = \frac{True\ Positives}{True\ Positives + True\ Negatives}$$

B.3.5 Precision

Precision indicates the accuracy of positive predictions made by the model. It is the ratio of true positive predictions to the total instances predicted as positive. The formula for precision is:

Eq. (B.3.5) $$Precision = \frac{True\ Positives}{True\ Positives + False\ Positives}$$

B.3.6 F_1-Score

The F1 score combines precision and recall into a single classification metric, ranging from 0 (low performance with no correct positive predictions) to 1 (perfect precision and recall). It corresponds to the harmonic mean of precision and recall. The formula for the F1 score is:

Eq. (B.3.6) $$F_1 = \frac{2 \times Precision \times Recall}{Precision + Recall} = \frac{True\ positives}{True\ positives + (False\ positives + False\ negatives)^{-2}}$$

B.3.7 ROC and ROC-AUC

The receiver operating characteristic (ROC) curve is a graphical representation of a model's performance across different classification thresholds. It plots the true positive rate against the false positive rate for various thresholds to visualize the trade-off between sensitivity and specificity.

The area under the ROC Curve (ROC-AUC) provides a quantitative measure of a model's ability to distinguish between classes across all possible thresholds. It is insensitive to class imbalance and computes the area under the ROC curve and ranges from 0 to 1. A higher AUC indicates better performance.

Eq. (B.3.7) $$ROC\ AUC = \int_0^1 Recall\, d(1 - \text{Specificity})$$

with $ROC\ AUC \in [0; 1]$

B.3.8 Matthew's correlation coefficient

The Matthews Correlation Coefficient (MCC), also referred to as the phi (φ) coefficient, offers a balanced assessment of binary classification performance based on label frequency distribution.

Eq. (B.3.8) $$MCC = \frac{TP \times TN - FP \times FN}{\sqrt{(TP+FP)(TP+FN)(TN+FP)(TN+FN)}}$$

with $MCC \in [-1; 1]$

B.4 Classifier model comparison

B.4.1 Cochran's Q test

The Cochran Q test [3] may be used to assess significant differences among the classifiers' performances [4]. The test statistic Q is computed by summing the squared differences between true positives for each classifier and the total number of true positives, normalized by subtracting the sum of squared differences for each sample from the product of the number of classifiers and the overall total of true positives. It follows a chi-square distribution with degrees of freedom equal to the number of classifiers minus one.

Eq. (B.4.1) $$Q = (L-1)\frac{L \times \sum_{i=1}^{L} TP_i^2 - {TP_{total}}^2}{L \times TP_{total} - \sum_{j=1}^{n} M_j^2}$$

with $TP_{total} = \left(\sum_{k=1}^{L} TP_k\right)^2$

where L represents the number of classifiers, TP_i the number of true positives for each i-th classifier, TP_{total} the total number of true positives among all classifiers, n the total number of EEG segments, and M_j the number of classifiers out of L that correctly predicted the class label of the j-th segment.

B.4.2 McNemar's test

The McNemar test [5] is a paired nonparametric statistical hypothesis test used to determine if there is a statistically significant difference in the model's misclassification errors, helping assess whether two models have significantly different predictive performance. McNemar's statistic X^2 and p-value was computed using a contingency table (see Table S5) that illustrates the correct and incorrect predicted labels of the classifiers:

Eq. (B.4.2) $X^2 = \frac{(|B - C| - 1)^2}{B+C}$ and

$$p = 2\sum_{i=B}^{n} \binom{n}{i} 0.5^i (1-0.5)^{n-i}$$

with $n = B + C$

Table S5
McNemar contingency table

		Model 2	
		correct	wrong
Model 1	correct	A	B
	wrong	C	D

A continuity corrected McNemar's test [6] was conducted using a chi-squared test with one degree of freedom.

B.5 References

1. Barandas, M. et al. TSFEL: Time Series Feature Extraction Library. SoftwareX **11**, 100456 (2020).

2. Pedregosa, F. et al. Scikit-learn: Machine Learning in Python. Journal of Machine Learning Research **12**, 2825–2830 (2011).

3. Cochran, W. G. THE COMPARISON OF PERCENTAGES IN MATCHED SAMPLES. Biometrika **37**, 256–266 (1950).

4. Dietterich, T. Approximate Statistical Tests for Comparing Supervised Classification Learning Algorithms. Neural Comput **10**, 1895–1923 (1998).

5. McNemar, Q. Note on the sampling error of the difference between correlated proportions or percentages. Psychometrika **12**, 153–157 (1947).

6. Edwards, A. L. Note on the “correction for continuity” in testing the significance of the difference between correlated proportions. Psychometrika **13**, 185–187 (1948).

C. Seizure quality indices

The following section elaborates on seizure quality indices, calculated in a manner comparable to those of the stimulation device [1,2]. In all calculations, the initial post-stimulation segment is excluded.

C.1 Seizure quality indices

C.1.1 Seizure Duration

The seizure duration is determined by the time lapse in seconds between the seizure onset and termination. In adherence to the seizure detection methodology detailed in section 2.5, seizures are determined by a minimum 8-second duration.

Eq. (C.1.1) $$Seizure\ Duration\ (s) = t_{seizure\ termination} - t_{seizure\ onset}$$

with $t_{seizure\ onset} < t_{seizure\ termination}$

where $t_{seizure\ onset}$ represents the timepoint of seizure onset and $t_{seizure\ termination}$ the timepoint of seizure conclusion.

C.1.2 Postictal Suppression Index

The Postictal Suppression Index (PSI) serves as a quantitative indicator for assessing the transition from seizure-associated spike-wave patterns to a subdued non-convulsive signal following the termination of a seizure event. The index is determined by comparing the average amplitudes of three postictal segments with those of three segments during the seizure. To mitigate the impact of artifacts, a time window equivalent to three segments around the seizure endpoint is disregarded. In case of more agitated postictal EEG, a negative PSI would be the result. In this analysis, these values were replaced by zero.

Eq. (C.1.2) $$PSI\ (\%) = \left(1 - \frac{\sum_{i=1}^{3} \left|A_{(STS\,+\,1)\,+\,i}\right|}{\sum_{i=1}^{3} \left|A_{(STS-\,1)\,-\,i}\right|}\right) \times 100$$

with $PSI \in [0; 100]$

where STS represents the segment immediately surrounding seizure termination, $\left|A_{(STS\,+\,1)\,+\,i}\right|$ defines the absolute EEG signal amplitude in the i-th postictal segment, and $\left|A_{(STS-\,1)\,-\,i}\right|$ denotes the absolute EEG signal amplitude in the i-th pre-terminal convulsive segment.

C.1.3 Average Seizure Energy Index

The Average Seizure Energy Index (ASEI) quantifies the average energy delivered during the induced ECT seizure, providing an indication of seizure intensity. It is calculated by the mean power of the EEG signal during the ictal period.

Eq. (C.1.3) $$\text{ASEI}\ (\mu V^2) = \frac{1}{Seizure\ Duration}\int_{t_{seizure\ onset}}^{t_{seizure\ termination}} P(t)dt$$

with $t_{seizure\ termination} > t_{seizure\ onset}$ and $P(t) = x(t)^2$

where $P(t)$ representing the power and $x(t)$ the EEG signal amplitude at time t. The $seizure\ Duration$ is defined as the time interval between $t_{seizure\ onset}$, the timepoint of seizure onset, and $t_{seizure\ termination}$, the timepoint of seizure conclusion (as defined in C.1.1).

C.1.4 Early-Ictal Amplitude

The Early-Ictal Amplitude (EIA) is a measure that characterizes the amplitude of brain signals during the initial phase of a seizure event. The EIA is calculated as the average absolute amplitude of the EEG signal over the very first eight seizure segments.

Eq. (C.1.4) $$\text{EIA}\ (\mu V) = \frac{1}{8}\sum_{i=SOS}^{SOS+7} |A_i|$$

with $SOS < (STS - 7)$

where SOS represents the seizure onset segment, STS the seizure termination segment, and $|A_i|$ the absolute amplitude of the EEG signal in the i-th initial ictal segment.

C.1.5 Mid-Ictal Amplitude

The mid-ictal amplitude (MIA) is a metric used to quantify the maximum average amplitude of EEG signals observed during the middle phase of a seizure episode. This metric is computed by evaluating the average absolute amplitudes across consecutive sets of eight segments throughout the seizure and selecting the maximum value.

Eq. (C.1.5) $$MIA\ (\mu V) = max_{SOS \leq k < (STS-8)}\left(\frac{1}{8}\sum_{i=0}^{7} |A_{k+i}|\right)$$

with $SOS < (STS - 7)$

where SOS represents the seizure onset segment, STS the seizure termination segment, k the starting segment index of each eight-segment window, i the segment index within that window, and $|A_{k+i}|$ the absolute amplitude of the $(k + i)$-th ictal segment.

C.1.6 Maximum Sustained Coherence

The Maximum Sustained Coherence (COH) assesses interhemispheric synchronization by measuring the highest mean coherence between EEG channels across eight-segment windows during a seizure.

Eq. (C.1.6) $$COH\ (\%) = max_{SOS \leq k < (STS-8)} \left(\frac{1}{8} \sum_{i=0}^{7} C_{k+i}\right)$$

with $C = \frac{|P_{left-right}|^2}{P_{left} \times P_{right}}$ and $SOS < (STS - 7)$

where SOS represents the seizure onset segment, STS the seizure termination segment, k the starting segment index of each eight-segment window, i the segment index within that window, and C_{k+i} the bifrontal signal coherence C of each $(k + i)$-th ictal segment with $P_{left-right}$ the cross spectral density of both hemispheric signals, and P_{left} and P_{right} the power spectral density estimates of the left and right frontal derivation, respectively [3].

C.1.7 Maximum Sustained Power

The Maximum Sustained Power (MSP) refers to the peak average signal power during a detected seizure over any eight consecutive segments.

Eq. (C.1.7) $$MSP\left(\frac{\mu V^2}{Hz}\right) = \max_{SOS \leq k < (STS-8)} \left(\frac{1}{8} \sum_{i=0}^{7} P_{k+i}\right)$$

with $SOS < (STS - 7)$

where SOS represents the seizure onset segment, STS the seizure termination segment, k the starting index of each eight-segment window during seizure, i the index within that window, and P_{k+i} corresponds to the average EEG power in the $(k + i)$-th segment.

C.1.8 Time To Peak Power

The Time To Peak Power (TTPP) corresponds to the duration from seizure onset to the midpoint of the time window with peak average power, denoted as the timepoint of maximum sustained power.

Eq. (C.1.8) $$TTPP\ (s) = t_{MSP} - t_{seizure\ onset}$$

with $t_{MSP} \geq (t_{seizure\ onset} + 5.12)$

where t_{MSP} represents the timepoint of maximum sustained power and $t_{seizure\ onset}$ the timepoint of seizure onset.

C.2 References

1. Somatics LLC. User Manual - Thymatron® System IV. (2021).
2. Abrams, R. & Swartz, C. M. Thymatron™ System IV Instruction Manual (5th ed., September 20, 2000).

3. Virtanen, P. et al. SciPy 1.0: fundamental algorithms for scientific computing in Python. Nat Methods **17**, 261–272 (2020).
